# Spontaneous Heterotopic Pregnancy in a Primigravida: A Case Report

**DOI:** 10.7759/cureus.118018

**Published:** 2026-10-16

**Authors:** Amr Elmoheen, Yaisel P Leon, Ahmed Elmesery, Matab Elbashir Hamad Hassan, Rehab Elmoheen

**Affiliations:** 1 Emergency Medicine, Qatar University Health Sector (QU Health), Doha, QAT; 2 Emergency Medicine, Hamad Medical Corporation, Doha, QAT; 3 Radiology, Sultan Qaboos Hospital, Salalah, OMN; 4 Emergency Medicine, Faculty of Medicine and Health Sciences, Al Neelain University, Khartoum, SDN; 5 Obstetrics and Gynecology, Ain Shams University, Cairo, EGY; 6 Obstetrics and Gynecology, Al-Qunfudhah General Hospital, Al-Qunfudhah, SAU

**Keywords:** ectopic pregnancy, emergency medicine, heterotopic pregnancy, intrauterine pregnancy, laparoscopic salpingectomy, pelvic ultrasound, spontaneous conception

## Abstract

Heterotopic pregnancy is the coexistence of an intrauterine pregnancy and an ectopic pregnancy. It is uncommon after spontaneous conception and may be overlooked once a normal intrauterine pregnancy has been identified. We report the case of a 33-year-old primigravida at approximately seven weeks of gestation who presented to the emergency department with sudden severe lower abdominal pain radiating to the back. Conception had occurred spontaneously, without assisted reproductive technology. Pelvic ultrasonography demonstrated a viable intrauterine pregnancy together with a separate viable left tubal pregnancy and free intraperitoneal fluid. Initial investigations showed microcytic anemia, with a hemoglobin concentration of 7.6 g/dL and a mean corpuscular volume of 66.6 fL. Laparoscopy demonstrated approximately 150 mL of hemoperitoneum and a macroscopically unruptured left tubal ectopic pregnancy with a fimbrial hematoma. A left salpingectomy was performed while preserving the intrauterine pregnancy. Histopathologic examination of the excised tube confirmed chorionic villi. Recovery was uncomplicated, and ultrasonography the following day confirmed continued viability of the intrauterine pregnancy. Subsequent first-trimester follow-up showed appropriate progression. The intrauterine pregnancy continued to term, with cesarean delivery of a live male infant at 39 weeks and four days for breech presentation in labor. This case emphasizes that identification of an intrauterine pregnancy should not end the evaluation of a pregnant patient with significant abdominal pain. Careful assessment of the adnexa remains important even after spontaneous conception.

## Introduction

Heterotopic pregnancy (HP) is defined by the simultaneous presence of an intrauterine and an extrauterine pregnancy. After spontaneous conception, it remains rare, with the incidence traditionally estimated at approximately one in 30,000 pregnancies [1]. Frequency is substantially higher after assisted reproductive technology (ART), and previous tubal disease, pelvic surgery, pelvic infection, and fertility treatment are recognized risk factors [1,2]. Spontaneous HP can also occur in patients without recognized risk factors [1].

The diagnostic difficulty is partly created by the intrauterine pregnancy itself. Once an apparently normal intrauterine gestation has been demonstrated, clinicians may be less likely to continue searching for an ectopic pregnancy. Recent reports of spontaneous HP continue to describe delayed or initially missed diagnoses after an intrauterine pregnancy had already been documented [3]. Persistent or significant abdominal pain, peritoneal findings, an adnexal abnormality, or unexplained intraperitoneal fluid should therefore prompt continued evaluation of the adnexa.

We present this case to highlight the importance of evaluating the adnexa in symptomatic patients despite a confirmed intrauterine pregnancy and the absence of recognized risk factors. Both pregnancies were viable at diagnosis. Early recognition allowed definitive treatment of the tubal pregnancy while preserving the intrauterine gestation.

## Case presentation

A 33-year-old primigravida presented to the emergency department (ED) with sudden-onset severe lower abdominal pain radiating to the back. Her last menstrual period was approximately 48 days prior to presentation, corresponding to approximately seven weeks of gestation. The pregnancy had occurred spontaneously, without ovulation induction or ART. The reported history did not suggest relevant medical or surgical risk factors, and there was no history of pelvic inflammatory disease, tubal pathology, or infertility. She denied nausea, vomiting, vaginal bleeding, vaginal discharge, and dysuria.

On arrival, the patient was alert and hemodynamically stable. Her temperature was 36.6°C, heart rate was 86 beats/minute, blood pressure was 101/69 mmHg, respiratory rate was 20 breaths/minute, and oxygen saturation was 96% on room air. Abdominal examination revealed generalized tenderness with rebound tenderness, most pronounced over the lower abdomen and both iliac fossae. Bedside ultrasonography demonstrated free intraperitoneal fluid.

Initial laboratory investigations showed a hemoglobin concentration of 7.6 g/dL and a hematocrit of 24.3%, with a mean corpuscular volume of 66.6 fL, mean corpuscular hemoglobin of 20.8 pg, and red cell distribution width of 17.2%. These findings demonstrated microcytic anemia; however, its duration and cause could not be established. Bedside transabdominal ultrasonography demonstrated an anechoic fluid collection in the hepatorenal recess (Morison’s pouch) on the right upper quadrant view, consistent with free intraperitoneal fluid (Figure 1).

**Figure 1:**
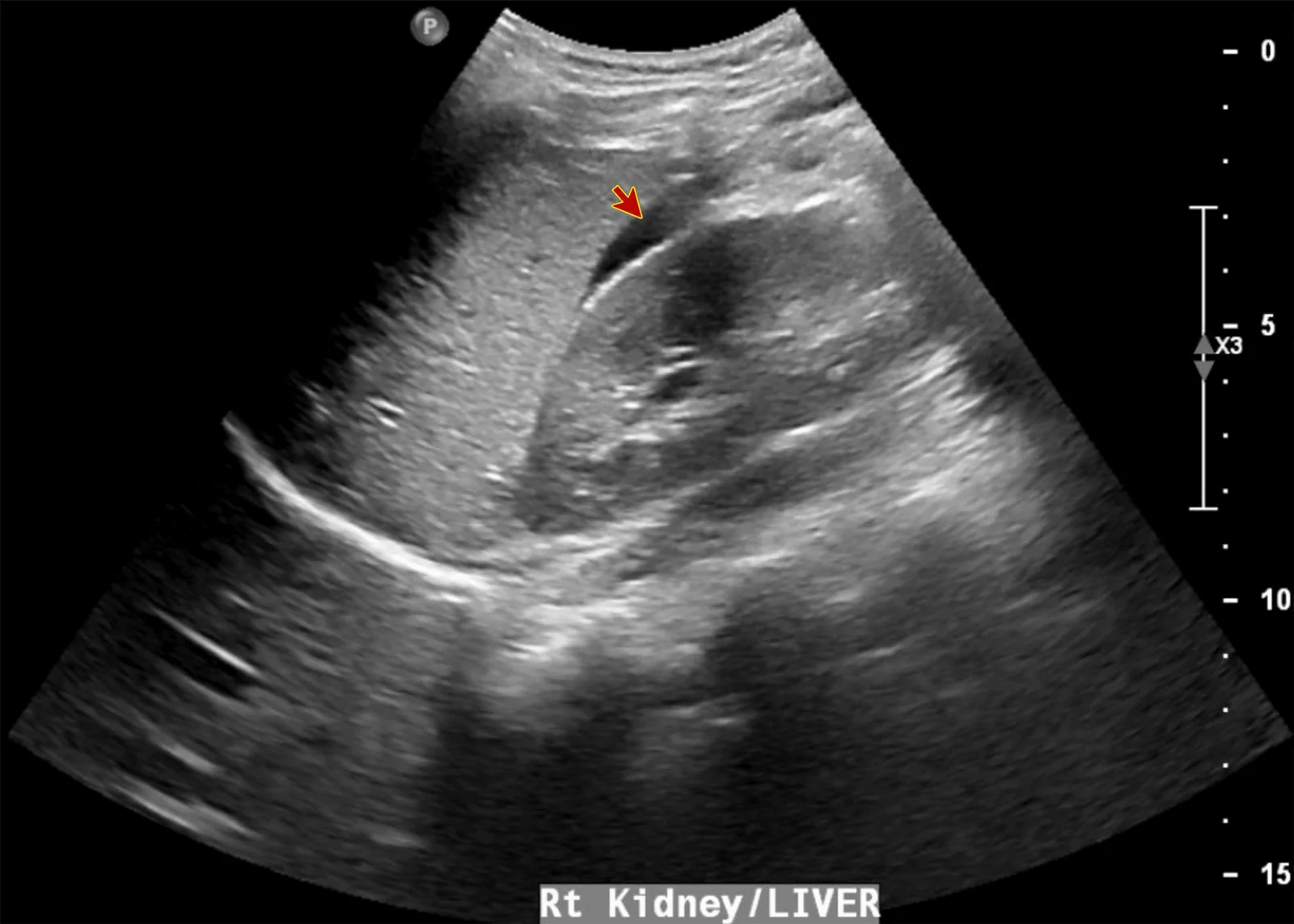
Bedside ultrasonography demonstrating free intraperitoneal fluid Right upper quadrant view showing an anechoic fluid collection in the hepatorenal recess (Morison’s pouch), indicated by the red arrow, consistent with free intraperitoneal fluid.

Formal transabdominal and transvaginal pelvic ultrasonography performed within one hour of arrival at the ED demonstrated a single live intrauterine pregnancy with a crown-rump length of approximately 11 mm, corresponding to approximately seven weeks of gestation. A separate gestational sac containing a fetal pole with cardiac activity and a crown-rump length of approximately 10 mm was identified in the left adnexa, also corresponding to approximately seven weeks of gestation and consistent with HP. Both ovaries were reported as unremarkable. Subsequent laparoscopy confirmed a left tubal ectopic pregnancy and a normal-appearing left ovary (Figures 2, 3).

**Figure 2:**
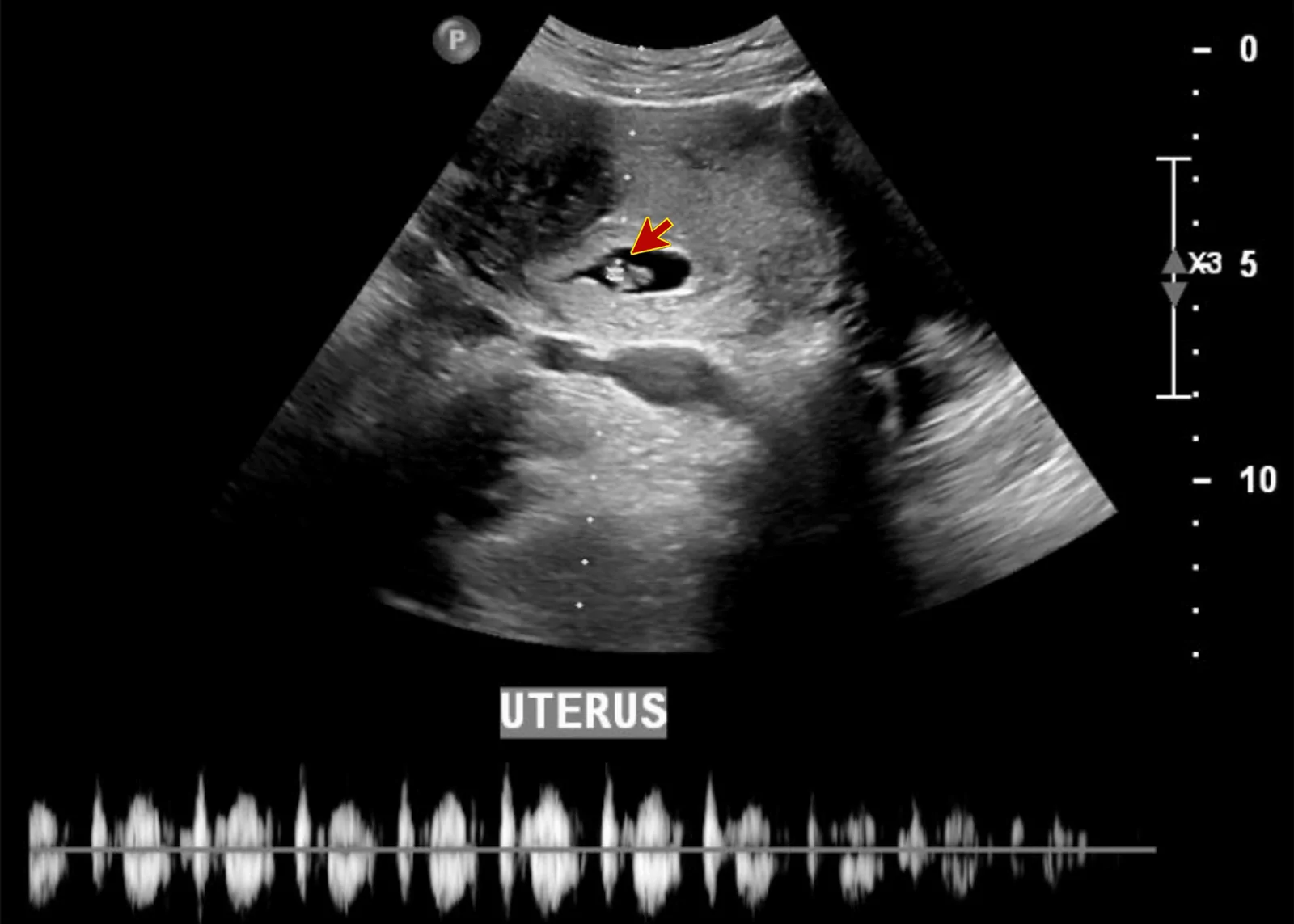
Doppler ultrasonography demonstrating a viable intrauterine pregnancy Pelvic ultrasound shows an intrauterine gestational sac containing a fetal pole (red arrow), with Doppler assessment confirming fetal cardiac activity, consistent with a live intrauterine pregnancy at approximately seven weeks of gestation.

**Figure 3:**
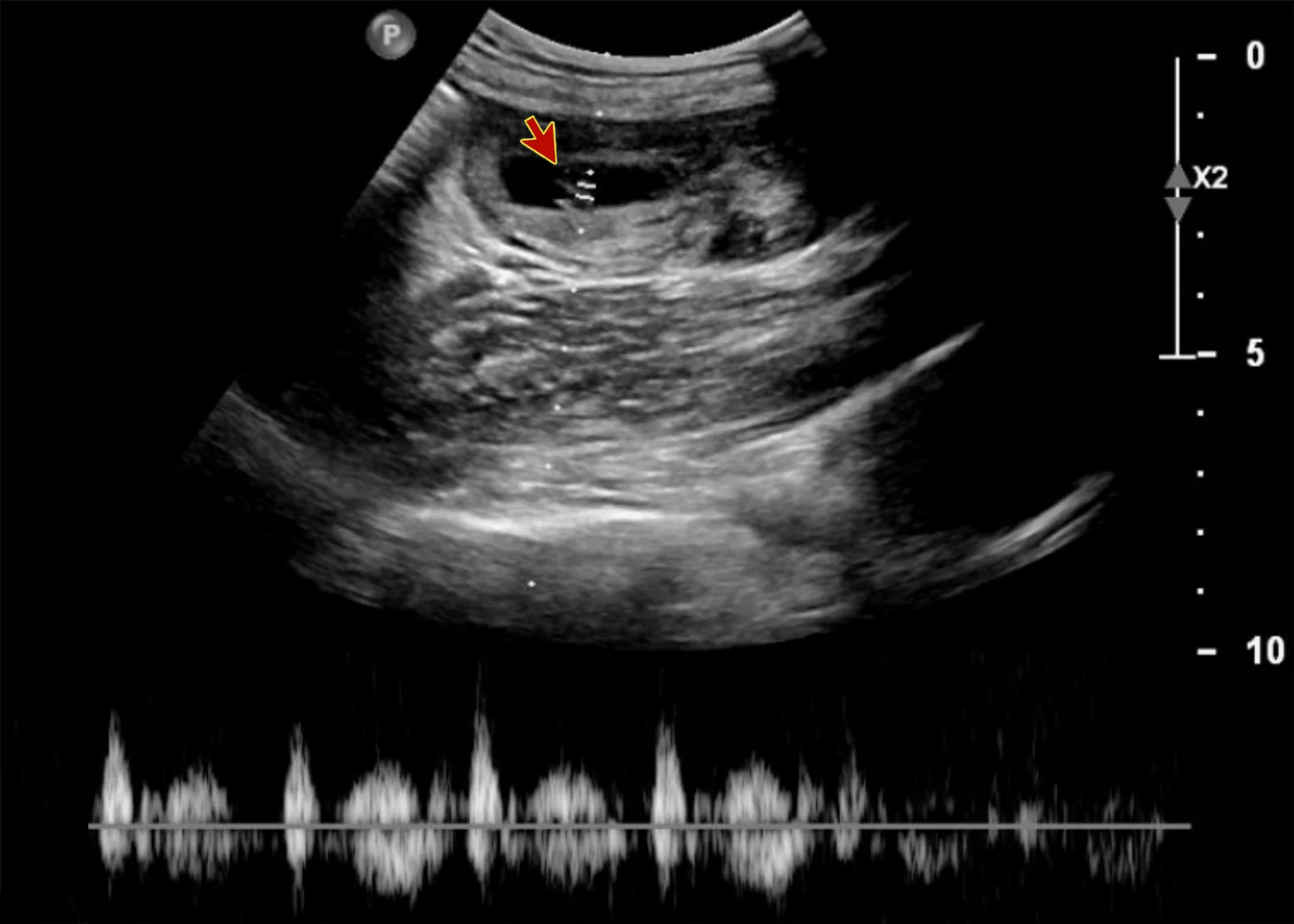
Doppler ultrasonography demonstrating a viable left tubal ectopic pregnancy Left adnexal ultrasound shows a separate extrauterine gestational sac containing a fetal pole (red arrow), with Doppler assessment confirming fetal cardiac activity, consistent with a live left tubal ectopic pregnancy at approximately seven weeks of gestation.

Moderate free fluid was present within the pelvis, raising concern for hemoperitoneum (Figure 4).

**Figure 4:**
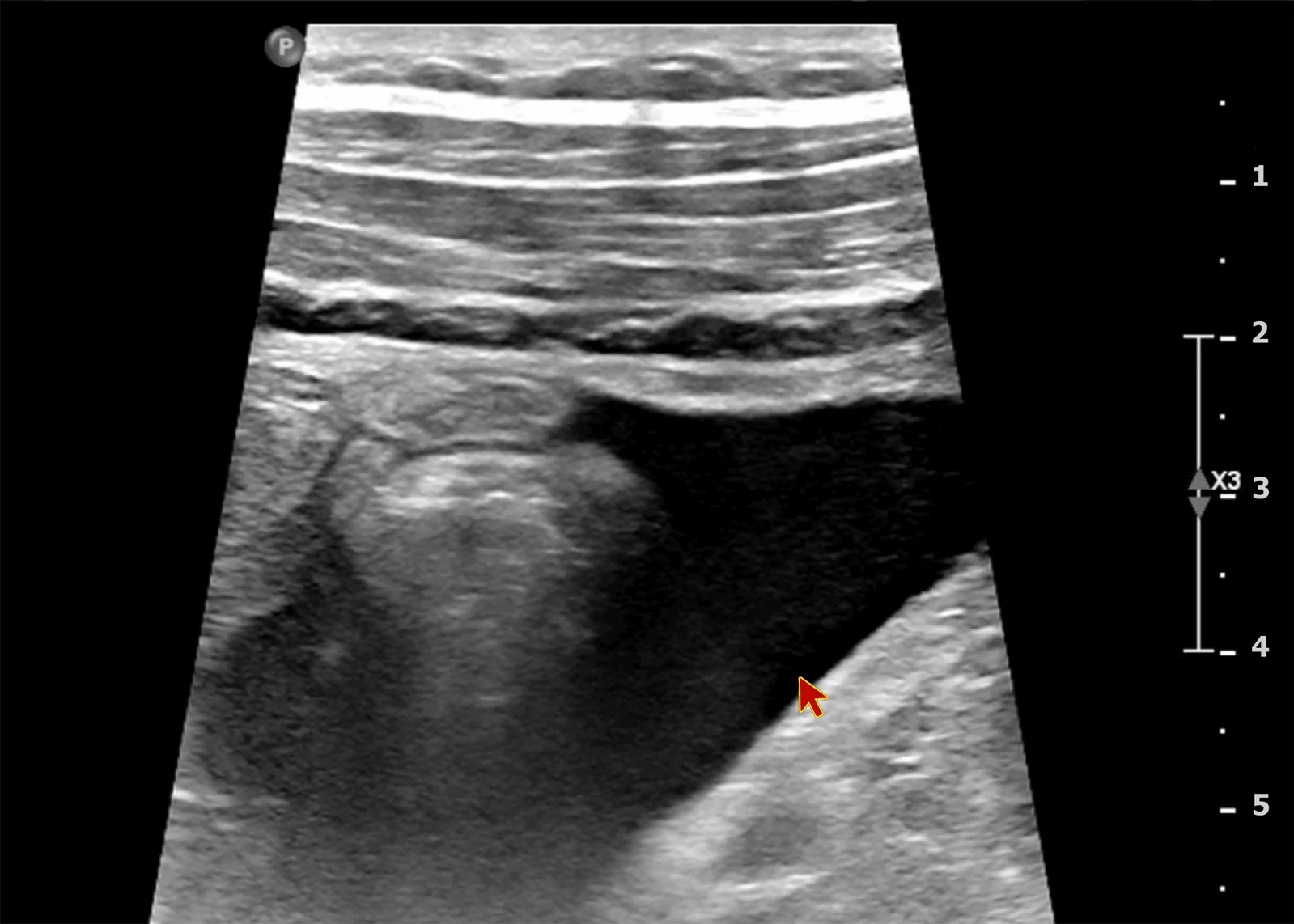
Pelvic ultrasonography demonstrating moderate free intraperitoneal fluid Anechoic free fluid is seen within the pelvis, indicated by the red arrow, consistent with hemoperitoneum in the clinical setting of heterotopic pregnancy.

The simultaneous presence of viable intrauterine and extrauterine pregnancies established the diagnosis of HP.

Initial management included intravenous fluids and paracetamol 1 g intravenously for analgesia. Two units of packed red blood cells were administered for marked anemia, with subsequent clinical improvement. Despite stable initial vital signs, severe abdominal pain, rebound tenderness, free intraperitoneal fluid, and ultrasonographic identification of a viable left extrauterine gestation prompted urgent gynecologic assessment and same-day laparoscopic management.

Laparoscopy demonstrated approximately 150 mL of hemoperitoneum and a macroscopically unruptured left tubal ectopic pregnancy measuring approximately 5 × 2 cm, with a 7 × 3 cm hematoma covering the fimbrial end. The left ovary appeared normal. Laparoscopic left salpingectomy was performed, and histopathological examination of the excised fallopian tube demonstrated chorionic villi, confirming the tubal ectopic gestation.

Her postoperative recovery was uncomplicated. Transvaginal ultrasonography performed on postoperative day 1 demonstrated a viable intrauterine pregnancy measuring seven weeks and one day, with documented fetal cardiac activity. Hemoglobin measured on the day of discharge was 8.5 g/dL. She remained clinically stable and was discharged with progesterone support for 14 days and iron supplementation. Follow-up during the 11- to 14-week period showed appropriate progression of the intrauterine pregnancy without further reported complications.

The intrauterine pregnancy continued to term. At 39 weeks and four days of gestation, the patient underwent a lower-segment cesarean section for breech presentation in labor. A live male infant was delivered with Apgar scores of 7 and 9 at one and five minutes, respectively. The mother was discharged in stable condition.

## Discussion

The diagnostic difficulty in this case was not the sonographic appearance once both pregnancies were visualized; it was the low initial likelihood of suspecting HP in a primigravida who had conceived naturally and had no recognized risk factors. In the systematic review by Oancea et al., abdominal pain was the most frequent presentation of spontaneous HP, and most reported patients had no identifiable predisposing factor [1]. More recently, Zhao et al. described the same diagnostic problem in a naturally conceived pregnancy, emphasizing that documentation of an intrauterine pregnancy can provide false reassurance and contribute to delayed recognition of the ectopic component [3]. Absence of vaginal bleeding does not exclude an ectopic component. Kudu et al. described an isolated ruptured ectopic pregnancy without abdominal pain or vaginal bleeding, in which marked anemia and free intraperitoneal fluid on bedside ultrasound prompted further evaluation [4]. Although this was not a HP, it illustrates the potential for atypical presentation to misdirect assessment. Our patient had severe abdominal pain without vaginal bleeding.

Ultrasonography was decisive. In practical terms, demonstration of an intrauterine pregnancy is not an exclusion test for a second ectopic gestation. When symptoms are disproportionate to the apparent intrauterine findings, the adnexa and pelvis should still be examined carefully. In our patient, the diagnosis became clear when a second gestational sac containing a fetal pole with cardiac activity was identified in the left adnexa. The accompanying free intraperitoneal fluid was particularly important in the context of severe pain and rebound tenderness [1,3].

Tubal ectopic pregnancies may also behave less predictably than their typical early presentation suggests. Elmoheen et al. previously reported an intact tubal ectopic pregnancy persisting into the 14th week of gestation, with fetal measurements corresponding to approximately 14 weeks and histopathologic confirmation of chorionic villi and gestational tissue within the fallopian tube [5]. In this case, laparoscopy demonstrated approximately 150 mL of hemoperitoneum and a macroscopically unruptured left tubal ectopic pregnancy, with a hematoma measuring approximately 7 × 3 cm covering the fimbrial end. Although the fimbrial hematoma was documented, active fimbrial bleeding or tubal abortion was not described. The operative appearance of an intact tube should therefore be interpreted alongside the patient's symptoms, imaging, and intraperitoneal findings rather than considered in isolation.

Management of HP differs from that of an isolated ectopic pregnancy because treatment of the ectopic component must take the desired intrauterine pregnancy into account. Systemic methotrexate should not be offered when a viable intrauterine pregnancy is intended to continue. The Society of Obstetricians and Gynaecologists of Canada guideline specifically advises against systemic methotrexate in the presence of a desired intrauterine pregnancy and recommends surgical excision of the ectopic component as an appropriate treatment for HP [6]. Despite hemodynamic stability, severe abdominal pain, rebound tenderness, hemoperitoneum, and a viable tubal gestation favored prompt operative management. Laparoscopic left salpingectomy was performed, and postoperative ultrasonography confirmed continued viability of the intrauterine pregnancy.

The available comparative evidence is reassuring regarding laparoscopy, although it should be interpreted cautiously. Zhou and Fu's systematic review and meta-analysis included 22 retrospective studies involving 1,133 patients with HP [7]. No statistically significant differences were found between laparoscopy and laparotomy in live birth, miscarriage, preterm birth, term birth, cesarean delivery, or vaginal delivery. All included studies were conducted in China, and the certainty of evidence was rated as low because the underlying studies were observational and some estimates were imprecise. The review found no statistically significant differences in reported pregnancy outcomes, but the low-certainty observational evidence does not establish equivalence between the approaches

Hu et al. reported 72 live births among 87 patients with ART-associated HP who underwent laparoscopic management, corresponding to a live birth rate of 82.76% [8]. These findings should be interpreted within the context of an ART population and cannot be directly extrapolated to spontaneous HP. In the present case, the spontaneously conceived intrauterine pregnancy continued after laparoscopic salpingectomy, culminating in the delivery of a live male infant by cesarean section at 39 weeks and four days for breech presentation in labor. Although this represents a favorable outcome, a single case cannot establish comparative treatment effectiveness or attribute the outcome solely to surgical management.

As with any single case, the findings cannot determine the relative value of different diagnostic or treatment strategies. The practical message is narrower. In a pregnant patient with substantial abdominal pain, peritoneal findings, or unexplained intraperitoneal fluid, the diagnostic evaluation should not stop simply because an intrauterine pregnancy has been demonstrated. Completing the adnexal examination may be the step that identifies the second pregnancy before significant deterioration occurs.

## Conclusions

Spontaneous HP should remain a diagnostic consideration in symptomatic patients despite a confirmed intrauterine pregnancy, spontaneous conception, and no recognized risk factors. Persistent or severe abdominal pain, peritoneal signs, adnexal abnormalities, or intraperitoneal fluid should prompt careful adnexal assessment. In this case, ultrasonography identified concurrent viable intrauterine and left extrauterine pregnancies. Laparoscopy demonstrated hemoperitoneum and a macroscopically unruptured left tubal ectopic pregnancy, which was treated by left salpingectomy. Histopathologic examination confirmed chorionic villi within the excised tube. The intrauterine pregnancy subsequently continued to term, with cesarean delivery of a live male infant at 39 weeks and four days for breech presentation in labor. This case highlights the importance of careful adnexal assessment in symptomatic patients despite a confirmed intrauterine pregnancy and documents a term live birth following surgical treatment of the ectopic component.
